# The Red Blood Cells on the Move!

**DOI:** 10.3389/fphys.2018.00474

**Published:** 2018-05-01

**Authors:** Anna Bogdanova, Lars Kaestner

**Affiliations:** ^1^Red Blood Cell Research Group, Institute of Veterinary Physiology, Vetsuisse Faculty and the Zurich Center for Integrative Human Physiology, University of Zurich, Zurich, Switzerland; ^2^Theoretical Medicine and Biosciences, Saarland University, Homburg/Saar and Experimental Physics, Saarland University, Saarbruecken, Germany

**Keywords:** erythrocyte, erythropiesis, personalized medicine, transfusion, modeling, point of care

Following the steps of the founders of modern red blood cell (RBC) research (e.g., Ørskov, 1935; Wilbrandt, 1937; Cohn and Cohn, 1939; Ponder, 1948; Pauling et al., 1949), we answer to the growing need of a platform to share and discuss the new findings, argue and move forward in our understanding of these amazing cells. We herewith initiate a new section of the Frontiers in Physiology dedicated to Red Blood Cell Physiology by naming some of the grand challenges in the areas that have to be addressed in the years to come from our point of view. These are basic problems related to our understanding of the structure and function of RBCs from a different perspective as well as the need of new approaches and solutions in translational hematological research.

## Basic science

In the clinical environment we still refer to “mean red blood cell volume” MCV, mean corpuscular hemoglobin concentration” MCHC and other “mean” values of RBC indices and characteristics such as deformability, density and longevity and others (Brugnara and Mohandas, 2013; Da Costa et al., 2016). We also operate with bulk responses of RBCs to stimuli comparing one “steady state” to the new one all cells miraculously acquire at the same time in our test tubes. Does it always make sense?

Form what we have learned about RBCs during the past decades multiple fractions of RBCs exist in our blood having distinct properties and sensitivity to stressors. The number of these fractions and the number of RBCs forming each of them varies between the healthy humans, and changes strikingly in patients with different diseases. We know close to nothing about the factors in control of this variability, only that it cannot be explained by the age of RBCs alone (Lutz and Bogdanova, 2013; Wang et al., 2013). This gap in knowledge demands more attention of researchers and the application of old (like density centrifugation or fluorescence based cell sorting) and new technological approaches (like single cell high throughput technologies) allowing to obtain readouts of subpopulations or even individual cells. Information on up to a few thousand RBCs will give the overview of the populations of cells forming fractions.

One more fundamental problem is to define a “steady state condition” we usually refer to, and whether it exists at all. Progress in live cell imaging revealed that RBCs are dynamic entities that choose one of several preferred states and may be found in any of those at any point in time when in flow. They move, Ca^2+^ levels in them oscillate, their ion content and pre-membrane ATP levels fluctuate too. Very little is known about the dynamics of these processes. We already started to farewell this snapshot-like approaches but there are still challenges to overcome.

The impact of RBC heterogeneity and its dynamic adaptive nature on blood rheology and gas exchange remains unclear. Addressing these questions is impossible without development of new integrative interdisciplinary experimental and theoretical modeling approaches.

A concept currently emerging, suggests that any stress conditions including acute or chronic stimulation of de novo RBC production affect signaling pathways engaged in control of proliferation and differentiation of erythroid precursor cells (Breda and Rivella, 2014). As a result, newly produced RBCs may differ in properties from those circulating in organisms with unstressed erythropoiesis. What are the mechanisms behind stress erythropoiesis and what are the properties of RBCs produced in response to stress as well as the role these cells play in adaptation to environmental challenge requires detailed investigation? Research in this area will have a vast impact at the translational level as stress erythropoiesis is acknowledged for patients presented with chronic hemolytic state.

Despite decades of studies we still lack basic understanding of the maintenance of RBC structure and function in the course of 100–120 days RBCs spend in the circulation (Vácha, 1983). Protein turnover in nucleated cells is typically in the range of hours to a couple of days, far shorter life span range than months that exact same proteins remain functional in RBCs. This impressive longevity of cells was not studied in details. Lacking translation, the maintenance of RBC function under the stressful conditions in the circulation is impressive and not fully understood (Kaestner and Minetti, 2017).

Furthermore, we would like to draw attention of researchers and readership of a new section in Frontiers to the role of experimental design in maintaining “physiological conditions.” To what extent shear, plasma constituents like albumin, amino acids and other factors that are usually neglected in the experimental settings impact the levels of NO and GSH production and Ca^2+^ signaling in RBCs is not clear. Do hormones, inflammatory factors, temperature and CO_2_ availability matter for mimicking the physiologically-relevant environment? We came to the point when standardized meaningful experimental conditions should be adapted for the majority of research work in order to better be able to compare and discuss the findings obtained by different groups. These requirements, however, should not hinder innovation. Some sensitive points in experimental design that help to avoid multiple artifacts have been discussed in the past (Minetti et al., 2013). Those include the need of leucodepletion to prevent proteolysis that may be detected using zymography. Meanwhile it has been shown that even zymography is not sensitive enough to avoid “contamination” with leucocytes as next generation sequencing of the transcriptome revealed the presence of traces of “contaminations” with leucocytes in RBC preparations obtained after filtration through the cellulose column (Danielczok et al., 2017).

For a long time, comparative approach to red cell biology was largely abandoned in the literature. As a result, all we know about RBCs of different species dates back to the reports of mid-to-end of twentieth century and earlier (e.g., Nikinmaa, 1990). Information on adaptation and resistance to infections as well as basic knowledge in comparative red cell biology may eventually teach us how to cure diseases from the evolution perspective. This knowledge is at least equally (if not more) valuable compared to that obtained from transgenic animals.

Several concepts within RBC research field are still awaiting strong experimental arguments to be proven or disapproved. Among them are neocytolysis (Risso et al., 2014) and the role of RBCs in systemic circadian rhythm maintenance (O'Neill and Reddy, 2011).

## Translational challenges

Modern world aims to provide optimal therapy to each of over 1.5 billion people suffering from anemia, be it caused by mutations or malnutrition of people (de Benoist et al., 2008). This incredible statistics stems from the lack of medical aid, but also from the insufficient understanding of the causes of disease and processes involved in its pathological manifestations. Gene editing therapy and bone marrow transplantation for treatment of patients with hereditary hemolytic anemias such as sickle cell disease or thalassemia are the ultimate treatments of choice. However, they are currently available to a small number of patients in developing countries, but not at locations where most of patients currently reside, in Asian and African countries. Both, diagnostic methods and therapeutic approaches for these cohorts of patients should be affordable, reliable and robust. Novel point of care (PoC) tests, e.g., for sickle cell disease screening or quantitative G6PD-deficiency tests, should address the needs of the patients in low income countries and/or in remote locations. New supportive therapies most likely will address the symptoms, not the mutations themselves. These therapies, as well as tests for responsiveness of patients to the new treatments are currently under development by translational researchers (biologists, chemists, pharmacologists, physicists) in collaboration with clinical hematologists and industrial experts. It's worthwhile to mention that the development of new technologies and tests will include state-of-the-art informatics approaches, such as machine learning techniques.

New methods to monitor efficacy of treatment will require little volumes of blood and deliver information on severity of disease based on analysis of blood fractions and individual cells. If this works out, personalized medication in terms of drug selection and dose might even be realized on a global level. These new technologies and assays should become part of a standard PoC test battery more efficient than currently available and as mentioned above, affordable for all patients.

Transfusion research faces its revival. In addition to improved preservation techniques, reduction of transfusion-related complications and increase in longevity to transfused RBCs in the circulation (Withanawasam and Wright, 2017), a new field has emerged. Stem cells are being used as a source of RBC cultivation *in vitro* (Douay and Giarratana, 2005). This is a very challenging task for interdisciplinary teams composed of biologists, engineers and specialists in blood banking. We reached a stage where *in vitro* grown reticulocytes can be generated in the milliliter range and currently first transfusion trials on humans are performed. However, to scale up and reach the level of medical transfusion volumes, the development of bioreactors for expansion and differentiation of erythroid precursors to reticulocytes and their maturation to RBCs are required.

With the increasing availability of next generation sequencing methods, an increasing number of diseases including rare diseases can be associated with particular proteins and mutations therein. Translational research is urgently required to explore the link (e.g., the molecular signaling) between the mutation and the symptoms of the patient. Although there is good progress, even for the longest known mutation based disease, sickle cell anemia (Pauling et al., 1949), the link between the mutation in the hemoglobin and the irregular vaso occlusive crises is not entirely clear.

Considering the tools that will help to tackle these challenges we should emphasize the priority of research using human blood samples over results obtained for the countless transgenic mouse models. The findings obtained for these models, although useful, cannot be directly translated to the mechanisms of RBC diseases in humans. So, “curing mice” should not be the ultimate goal as long as RBCs of human (and other animal) patients are neglected.

Furthermore, to understand blood flow, that is different from Newtonian fluids and that is largely determined by the properties of RBCs, especially the formation of turbulences in pipe like structures will allow to capture the formation of vortices under physiological or pathological conditions, e.g., during atrial fibrillation (Park et al., 2013). Such questions can only be solved on an interdisciplinary base spanning from theoretical and experimental physics to biomedical and clinical research.

Our optimism in finding solutions to these problems relies on the revival of the interest to RBC research, impressive efficacy and devotion of the groups of scientists and clinical hematologists. The European Hematological Association and American Hematological Society, the American Red Cell Club and the European Red Cell Society (ERCS), as well as the European Network for Rare Congenital Anemias (ENERCA) work together on these challenging topics. Several European consortia receive funding from the EU to provide a breakthrough in generation of new knowledge, development of new technologies, studying red cell disorders and educating young scientists in these areas. Also programs or dedicated research institutions on the national level like the Laboratory of Excellence (GR-Ex) in France or Sanquin Research in The Netherlands are vital for future RBC research. We hope that a new information exchange platform within Frontiers in Physiology, the Red Blood Cell Physiology Section will contribute to the intensive and fast information exchange, promote discussions and education in red cell research. We invite all the experts and young researchers to join this platform with their best high quality red cell research and we promise a fair and transparent review process.

It's almost obvious that the RBC moves but we believe that after passing a period of contempt at the eve of the new millennium, RBCs are back to move biomedical science forward again.

## Author contributions

Both authors have made a substantial, direct and intellectual contribution to the work, and approved it for publication.

### Conflict of interest statement

The authors declare that the research was conducted in the absence of any commercial or financial relationships that could be construed as a potential conflict of interest. The reviewer JE and handling Editor declared their shared affiliation.
